# Supplementary material for: Altered microRNA Transcriptome in Cultured Human Liver Cells upon Infection with Ebola Virus
Source: Int J Mol Sci. 2021 Apr 6;22(7):3792. doi: 10.3390/ijms22073792 (PMC8038836; doi:10.3390/ijms22073792)
Supplement: Supplementary file 1 [file ijms-22-03792-s001.zip › Supplementary_File/C_ GO_Analysis_Results/16-30nt_go_Reston-96h-Huh7_vs_Control-96h-Huh7_down.mature_mirna_targets/CC_result(Human).html]

| GO.ID | Term | Ontology | Count | Pop.Hits | List.Total | Pop.Total | Fold.Enrichment | Pvalue | FDR | Enrichment.Score | Gene.Ratio | GENES |
| --- | --- | --- | --- | --- | --- | --- | --- | --- | --- | --- | --- | --- |
| GO:0045202 | synapse | Cellular component | 36 | 880 | 381 | 18698 | 2.00765926986399 | 5.87561270490353e-05 | 0.0504715131351213 | 4.23094683876106 | 0.094488188976378 | LRRTM1//BSN//ACTR2//CHRM1//CHRNA3//FBXO45//CPEB3//GRIN2B//PALM//RTN4//TMEM108//ITGA8//COPS5//STON2//ATP6V1G2//LRRK2//ABCC8//SCAMP1//CPLX1//AP1S1//PPP3CA//PRKAR2B//ZNF804A//ELK1//KCNIP3//SSPN//FZD3//P2RX2//GRIPAP1//LYPD6//CADM2//ITGA5//VWC2//CNTN2//NDFIP1//VAMP3// |
| GO:0030054 | cell junction | Cellular component | 44 | 1247 | 381 | 18698 | 1.73163518954678 | 0.000266724408648389 | 0.0887971621810643 | 3.57393723906734 | 0.115485564304462 | ACTR3//ITGA5//SKAP1//EIF4G2//CADM2//JAM3//CTNND2//CLDN18//USP53//PARD6B//CGNL1//SCN1A//ACTR2//FERMT2//FZD2//PDIA3//CD46//PARVA//RRAS//TLN1//YWHAB//YWHAZ//NUP214//ITGA8//DIXDC1//CD81//COPS5//CHRM1//CHRNA3//LRRK2//LYPD6//FBXO45//CPEB3//GRIN2B//LRRTM1//VWC2//GRIPAP1//TMEM108//NDFIP1//SSPN//FERMT3//STON2//BSN//VAMP3// |
| GO:0034703 | cation channel complex | Cellular component | 13 | 217 | 381 | 18698 | 2.94004378484947 | 0.000532710565765426 | 0.0887971621810643 | 3.27350868934598 | 0.0341207349081365 | GRIN2B//VWC2//SESTD1//KCNIP2//TRDN//LRRC38//KCNIP3//ABCC8//CNTN2//KCNQ4//SCN3B//SCN1A//SCN7A// |
| GO:0044456 | synapse part | Cellular component | 29 | 740 | 381 | 18698 | 1.92325317443428 | 0.000621837346587593 | 0.0887971621810643 | 3.20632319845583 | 0.0761154855643045 | COPS5//STON2//LRRK2//ABCC8//SCAMP1//FZD3//BSN//ZNF804A//P2RX2//PALM//ITGA8//FBXO45//CHRM1//CHRNA3//CPEB3//GRIN2B//LRRTM1//TMEM108//SSPN//ATP6V1G2//CPLX1//AP1S1//ELK1//KCNIP3//PPP3CA//PRKAR2B//GRIPAP1//ACTR2//RTN4// |
| GO:0030425 | dendrite | Cellular component | 24 | 569 | 381 | 18698 | 2.06999432628039 | 0.000666510989231864 | 0.0887971621810643 | 3.17619268567486 | 0.062992125984252 | LRRK2//PPP3CA//PRKAR2B//ZNF804A//CPEB3//RTN4//PALM//ITGA8//CPLX1//CHRM1//CHRNA3//CTNND2//ELK1//GNAO1//GNAZ//KCNIP3//GRIPAP1//PURA//RBM3//TMEM108//MLPH//FZD3//NDFIP1//BSN// |
| GO:0097447 | dendritic tree | Cellular component | 24 | 570 | 381 | 18698 | 2.06636275728692 | 0.000683019705661748 | 0.0887971621810643 | 3.1655667663965 | 0.062992125984252 | CPLX1//CHRM1//CHRNA3//LRRK2//CTNND2//ELK1//CPEB3//GNAO1//GNAZ//KCNIP3//GRIPAP1//PURA//RBM3//TMEM108//MLPH//FZD3//NDFIP1//BSN//PPP3CA//PRKAR2B//ZNF804A//RTN4//PALM//ITGA8// |
| GO:0005829 | cytosol | Cellular component | 130 | 5022 | 381 | 18698 | 1.27038928974977 | 0.000951125289540891 | 0.0887971621810643 | 3.02176227068375 | 0.341207349081365 | UCHL5//ACTR3//ACTR2//FAM13A//CALCOCO2//CDKN1B//EMC8//TRDN//CPLX1//RALBP1//TOMM34//FERMT2//COPS5//NISCH//AKAP10//AP1S1//LRRK2//UBXN2B//CTH//PNPLA5//RNF217//ARHGAP36//UBXN2A//DPYS//EIF4A2//EIF4G2//EMP2//EPAS1//HIPK1//ALAD//FABP2//MAPRE1//RRP1B//MGRN1//DDAH1//CARHSP1//ARFIP2//FTSJ1//SULT4A1//FBXL3//PTPN18//GNAZ//WDR91//KCNIP3//HIF1A//IDI1//KIF2A//KRT2//TNFAIP8L3//RHOH//SMAD1//NCAM1//NOS3//SPRN//PABPC3//PAX5//ABHD5//RLIM//UPB1//ATP6V1G2//PMAIP1//POLR2E//RHOF//SHQ1//RIC8B//NUDT15//VPS53//PPP3CA//RFK//PPP3CB//PRKAA1//MAP7D1//PARVA//PRKAR2B//BRK1//MAPK4//KLHL7//GRIPAP1//TULP4//PSMD7//KLHL14//WDFY1//PEX5//TP53INP2//PCTP//RPE//SFRP1//EPS8L2//CEP85//SKP1//HSPA13//TLN1//UBE2D3//UBE2E1//UCK2//BEST1//YWHAB//YWHAZ//RPAP3//ZNF322//PIP4K2C//AGBL2//NUP214//TNKS2//KRTAP1-1//CASP7//MEX3B//RNF135//PARD6B//CREB3L3//OGT//PARG//STON2//DIXDC1//SKAP1//ALDH1A2//KBTBD6//SYS1//CH25H//NFS1//BUB3//COPS2//VAMP3//PAXBP1//ARHGEF6//BAG5//BAG2//GOSR2//SEPT7//OXSR1// |
| GO:1902495 | transmembrane transporter complex | Cellular component | 16 | 321 | 381 | 18698 | 2.44616151952968 | 0.000959590933186412 | 0.0887971621810643 | 3.01791386416993 | 0.041994750656168 | CHRNA3//BEST1//GRIN2B//VWC2//SESTD1//KCNIP2//SCN3B//SCN1A//SCN7A//TRDN//LRRC38//KCNIP3//ABCC8//CNTN2//KCNQ4//ATP1B4// |
| GO:0036477 | somatodendritic compartment | Cellular component | 29 | 766 | 381 | 18698 | 1.85797304057619 | 0.00106377683947982 | 0.0887971621810643 | 2.97314946936006 | 0.0761154855643045 | CPLX1//CHRNA3//LRRK2//ELK1//PRKAR2B//RTN4//KLHL14//PURA//SCN1A//CNTN2//FZD3//POLR2M//ZNF804A//UNC5A//CTNND2//ITGA8//CHRM1//CPEB3//GNAO1//GNAZ//KCNIP3//GRIPAP1//RBM3//TMEM108//MLPH//NDFIP1//BSN//PPP3CA//PALM// |
| GO:0120025 | plasma membrane bounded cell projection | Cellular component | 60 | 1980 | 381 | 18698 | 1.48715501471407 | 0.00111485829584973 | 0.0887971621810643 | 2.9527803301418 | 0.15748031496063 | ARFIP2//ITGA5//TLN1//UBXN10//EVC//TULP4//CHRNA3//LRRK2//LYPD6//CPEB3//GRIN2B//NPTX1//KLHL14//STON2//VAMP3//TNFRSF8//ACTR3//ACTR2//BRK1//ARHGEF6//SCN7A//HIF1A//CCDC103//SEPT7//FAM168B//CADM2//LRRTM1//PALM//GRIPAP1//TMEM108//FZD3//BSN//CPLX1//CHRM1//CTNND2//ELK1//GNAO1//GNAZ//KCNIP3//PURA//RBM3//MLPH//NDFIP1//SLC27A4//AOC3//PPP3CA//PRKAR2B//ZNF804A//SH3YL1//EPS8L2//UNC5A//FERMT2//KIF2A//ITGA8//MAPRE1//SCN1A//CNTN2//AGBL2//AP1S1//RTN4// |
| GO:0034702 | ion channel complex | Cellular component | 15 | 296 | 381 | 18698 | 2.48696531176846 | 0.00116261777570612 | 0.0887971621810643 | 2.93456304123507 | 0.0393700787401575 | CHRNA3//BEST1//GRIN2B//VWC2//SESTD1//KCNIP2//SCN3B//SCN1A//SCN7A//TRDN//LRRC38//KCNIP3//ABCC8//CNTN2//KCNQ4// |
| GO:1990351 | transporter complex | Cellular component | 16 | 329 | 381 | 18698 | 2.38668038835571 | 0.00124047257994502 | 0.0887971621810643 | 2.90641283115814 | 0.041994750656168 | CHRNA3//GRIN2B//VWC2//SESTD1//KCNIP2//BEST1//ATP1B4//SCN3B//SCN1A//SCN7A//TRDN//LRRC38//KCNIP3//ABCC8//CNTN2//KCNQ4// |
| GO:0042995 | cell projection | Cellular component | 61 | 2055 | 381 | 18698 | 1.45676060565422 | 0.00166165005776605 | 0.109796723047772 | 2.77946043300876 | 0.16010498687664 | ARFIP2//ITGA5//TLN1//UBXN10//EVC//TULP4//CHRNA3//LRRK2//LYPD6//CPEB3//GRIN2B//NPTX1//KLHL14//STON2//VAMP3//TNFRSF8//ACTR3//ACTR2//BRK1//ARHGEF6//SCN7A//HIF1A//CCDC103//SEPT7//FAM168B//CADM2//LRRTM1//PALM//GRIPAP1//TMEM108//FZD3//BSN//CPLX1//CHRM1//CTNND2//ELK1//GNAO1//GNAZ//KCNIP3//PURA//RBM3//MLPH//NDFIP1//PPP3CA//PRKAR2B//ZNF804A//SLC27A4//AOC3//SH3YL1//EPS8L2//UNC5A//FERMT2//KIF2A//ITGA8//MAPRE1//SCN1A//CNTN2//AGBL2//AP1S1//RTN4//FERMT3// |
| GO:0097458 | neuron part | Cellular component | 48 | 1533 | 381 | 18698 | 1.53662983907833 | 0.00189247234348251 | 0.11565958234077 | 2.72297045854424 | 0.125984251968504 | COPS5//STON2//ACTR2//CHRM1//CHRNA3//FBXO45//CPEB3//GRIN2B//PALM//RTN4//TMEM108//ITGA8//BSN//LRRK2//LYPD6//NPTX1//KLHL14//VAMP3//TNFRSF8//ABCC8//SCAMP1//EPS8L2//UNC5A//CPLX1//ELK1//PRKAR2B//PURA//SCN1A//CNTN2//FZD3//POLR2M//ZNF804A//CTNND2//ACTL6A//FAM168B//CADM2//LRRTM1//GRIPAP1//GNAO1//GNAZ//KCNIP3//RBM3//MLPH//NDFIP1//AP1S1//HIF1A//ATP6V1G2//PPP3CA// |
| GO:0031674 | I band | Cellular component | 9 | 138 | 381 | 18698 | 3.20061622731941 | 0.00210344894930134 | 0.11565958234077 | 2.67706802384464 | 0.0236220472440945 | BMP10//PPP3CA//PPP3CB//PARVA//SCN3B//JPH1//SCN1A//FERMT2//POLR2M// |
| GO:0043005 | neuron projection | Cellular component | 40 | 1224 | 381 | 18698 | 1.60379462371125 | 0.00215431119610282 | 0.11565958234077 | 2.66669156148914 | 0.10498687664042 | LRRK2//FAM168B//CADM2//LRRTM1//PALM//GRIPAP1//TMEM108//FZD3//BSN//CPLX1//CHRM1//CHRNA3//CTNND2//ELK1//CPEB3//GNAO1//GNAZ//KCNIP3//PURA//RBM3//MLPH//NDFIP1//UNC5A//PPP3CA//PRKAR2B//ZNF804A//EPS8L2//ITGA8//HIF1A//SCN1A//CNTN2//AP1S1//RTN4//LYPD6//GRIN2B//NPTX1//KLHL14//STON2//VAMP3//TNFRSF8// |
| GO:0098794 | postsynapse | Cellular component | 19 | 450 | 381 | 18698 | 2.07210265383494 | 0.00233823545368382 | 0.118149662042024 | 2.6311117587486 | 0.0498687664041995 | PPP3CA//PRKAR2B//ZNF804A//CHRM1//CHRNA3//FBXO45//CPEB3//GRIN2B//LRRTM1//TMEM108//SSPN//PALM//ITGA8//GRIPAP1//ACTR2//RTN4//BSN//LRRK2//P2RX2// |
| GO:0001518 | voltage-gated sodium channel complex | Cellular component | 3 | 14 | 381 | 18698 | 10.5163104611924 | 0.00258523529851931 | 0.123373173412672 | 2.58749992289964 | 0.0078740157480315 | SCN3B//SCN1A//SCN7A// |
| GO:0005938 | cell cortex | Cellular component | 14 | 296 | 381 | 18698 | 2.32116762431723 | 0.00315247006630438 | 0.139679902153698 | 2.50134902849812 | 0.036745406824147 | BSN//MAEA//SEPT7//ACTR2//MLPH//POLR2M//MAPRE1//FERMT2//ARFIP2//RHOF//RIC8B//BFSP1//NDFIP1//PARD6B// |
| GO:0005737 | cytoplasm | Cellular component | 261 | 11544 | 381 | 18698 | 1.1095691390967 | 0.00325215138891032 | 0.139679902153698 | 2.48782924603059 | 0.68503937007874 | FERMT2//ARFIP2//RHOF//RIC8B//BFSP1//NDFIP1//PARD6B//LRRK2//HIF1A//TMEM108//TRDN//JPH1//CARHSP1//CCDC103//SEPT7//BSN//KDELR3//AP1S1//EMP2//GOLGA7B//NCAM1//NOS3//NRAS//ST3GAL3//SLC35A2//TNKS2//CREB3L3//GAL3ST1//GOSR2//PPP1R2//CEP85//CALCOCO2//PYURF//PNRC1//MEX3B//VPS53//SKIL//CD46//CTNS//DRAM1//SMPD1//TIMM23//EMC8//ZBTB6//TOMM34//AKAP10//STARD4//PPTC7//DLAT//ELK1//DDAH1//MYCBP//SLC25A5//ACADL//ME2//UCHL5//MRPS33//PMAIP1//PPP3CA//RFK//CREBZF//ABCC8//YWHAB//YWHAZ//OGT//NFS1//SH3BP5//BAG5//ACTR3//ACTR2//FAM13A//CDKN1B//CPLX1//RALBP1//COPS5//NISCH//UBXN2B//CTH//PNPLA5//RNF217//ARHGAP36//UBXN2A//DPYS//EIF4A2//EIF4G2//EPAS1//HIPK1//ALAD//FABP2//MAPRE1//RRP1B//MGRN1//FTSJ1//SULT4A1//FBXL3//PTPN18//GNAZ//WDR91//KCNIP3//IDI1//KIF2A//KRT2//TNFAIP8L3//RHOH//SMAD1//SPRN//PABPC3//PAX5//ABHD5//RLIM//UPB1//ATP6V1G2//POLR2E//SHQ1//NUDT15//PPP3CB//PRKAA1//MAP7D1//PARVA//PRKAR2B//BRK1//MAPK4//KLHL7//GRIPAP1//TULP4//PSMD7//KLHL14//WDFY1//PEX5//TP53INP2//PCTP//RPE//SFRP1//EPS8L2//SKP1//HSPA13//TLN1//UBE2D3//UBE2E1//UCK2//BEST1//RPAP3//ZNF322//PIP4K2C//AGBL2//NUP214//KRTAP1-1//CASP7//RNF135//PARG//STON2//DIXDC1//SKAP1//ALDH1A2//KBTBD6//SYS1//CH25H//BUB3//COPS2//VAMP3//PAXBP1//ARHGEF6//BAG2//OXSR1//SNN//TOMM40L//LETM2//GPD2//MRPL50//AADAT//VLDLR//SNX31//NUCB1//AOC3//NRBF2//PDIA3//LRRTM1//ITGA5//MEST//DNAJB12//RTN4//MOGAT2//TXNDC5//CAMLG//ITGA8//SPCS2//EBI3//DBI//GPX8//SLC27A4//SLC35G1//CYP4F3//INSIG2//OTOR//CYP4F2//JAM3//RBFOX1//SCAMP1//NPTX1//SSPN//ABCA13//MAEA//MRC1//LARP1//RBM3//BMP10//SCN3B//SCN1A//FZD2//MLPH//POLR2M//FERMT3//SLC39A2//PALM//CXXC4//TSPAN14//FAM168B//AVL9//UBE4B//PPIH//PRDM4//SLAIN1//LYPD6//NEK7//CTNND2//PRUNE2//DLX5//EVC//EWSR1//FAM83B//CPEB3//TTC28//CAPN7//HEYL//GDF9//KCNIP2//HMGB3//HMGN2//TLX2//MARK1//MEA1//MLF1//FOXP3//PFDN1//RALGPS2//STRBP//KMT2E//BEX4//PAPOLB//PURA//TTK//FZD3//TRIM7//SH3BGRL3//BHLHE40//TNFSF14//EMILIN3//ZNF804A//NREP//TNFRSF8//SLC23A2// |
| GO:0098797 | plasma membrane protein complex | Cellular component | 22 | 574 | 381 | 18698 | 1.88096609874985 | 0.00359720159967674 | 0.147142674958206 | 2.44403522212764 | 0.057742782152231 | GNAO1//GNAZ//SCN3B//SCN1A//SCN7A//ATP1B4//TRDN//LRRC38//KCNIP3//KCNIP2//ABCC8//CNTN2//KCNQ4//SSPN//ITGA5//ITGA8//CHRNA3//SKAP1//CD8A//GRIN2B//VWC2//EVC// |
| GO:0098590 | plasma membrane region | Cellular component | 35 | 1069 | 381 | 18698 | 1.60679517492493 | 0.00392608022582766 | 0.153295586999362 | 2.40604083023823 | 0.0918635170603675 | KCNQ4//SLC23A2//SLC26A1//PALM//BEST1//CD81//EMP2//P2RX2//PRKAA1//FZD3//PARD6B//CYP4F2//VAMP3//SEPT7//MAPRE1//CHRNA3//CD8A//LRRK2//FERMT2//SLC27A4//SH3YL1//ITGA5//EPS8L2//TLN1//UNC5A//EVC//NOS3//ITGA8//FBXO45//CHRM1//CPEB3//GRIN2B//LRRTM1//TMEM108//SSPN// |
| GO:0044463 | cell projection part | Cellular component | 42 | 1377 | 381 | 18698 | 1.4968749821305 | 0.00573132667532886 | 0.205133733921145 | 2.24174483683114 | 0.110236220472441 | CPLX1//CHRM1//CHRNA3//LRRK2//CTNND2//ELK1//CPEB3//GNAO1//GNAZ//KCNIP3//GRIPAP1//PURA//RBM3//TMEM108//MLPH//FZD3//NDFIP1//BSN//MAPRE1//EPS8L2//PPP3CA//PRKAR2B//ZNF804A//FERMT2//SLC27A4//PALM//SH3YL1//ITGA5//TLN1//UNC5A//ITGA8//EVC//HIF1A//LRRTM1//SCN1A//CNTN2//AP1S1//RTN4//CCDC103//SEPT7//AGBL2//KIF2A// |
| GO:0120038 | plasma membrane bounded cell projection part | Cellular component | 42 | 1377 | 381 | 18698 | 1.4968749821305 | 0.00573132667532886 | 0.205133733921145 | 2.24174483683114 | 0.110236220472441 | CPLX1//CHRM1//CHRNA3//LRRK2//CTNND2//ELK1//CPEB3//GNAO1//GNAZ//KCNIP3//GRIPAP1//PURA//RBM3//TMEM108//MLPH//FZD3//NDFIP1//BSN//MAPRE1//EPS8L2//PPP3CA//PRKAR2B//ZNF804A//FERMT2//SLC27A4//PALM//SH3YL1//ITGA5//TLN1//UNC5A//EVC//LRRTM1//SCN1A//CNTN2//AP1S1//HIF1A//RTN4//CCDC103//SEPT7//AGBL2//KIF2A//ITGA8// |
| GO:0005925 | focal adhesion | Cellular component | 16 | 393 | 381 | 18698 | 1.99800979076089 | 0.00704503015414309 | 0.222988489458029 | 2.1521171436831 | 0.041994750656168 | ACTR3//ACTR2//FERMT2//FZD2//PDIA3//ITGA5//CD46//PARVA//RRAS//TLN1//YWHAB//YWHAZ//NUP214//ITGA8//DIXDC1//CD81// |
| GO:0014069 | postsynaptic density | Cellular component | 11 | 227 | 381 | 18698 | 2.37813775480708 | 0.00705901770957125 | 0.222988489458029 | 2.15125572855064 | 0.0288713910761155 | GRIN2B//ACTR2//CHRM1//CHRNA3//FBXO45//CPEB3//PALM//RTN4//TMEM108//ITGA8//BSN// |
| GO:0099572 | postsynaptic specialization | Cellular component | 11 | 229 | 381 | 18698 | 2.35736799275636 | 0.00751845434147728 | 0.222988489458029 | 2.12387143333963 | 0.0288713910761155 | ACTR2//CHRM1//CHRNA3//FBXO45//CPEB3//GRIN2B//PALM//RTN4//TMEM108//ITGA8//BSN// |
| GO:0005924 | cell-substrate adherens junction | Cellular component | 16 | 396 | 381 | 18698 | 1.98287335295209 | 0.00755178803735068 | 0.222988489458029 | 2.12195020826254 | 0.041994750656168 | ACTR3//ACTR2//FERMT2//FZD2//PDIA3//ITGA5//CD46//PARVA//RRAS//TLN1//YWHAB//YWHAZ//NUP214//ITGA8//DIXDC1//CD81// |
| GO:0099568 | cytoplasmic region | Cellular component | 18 | 468 | 381 | 18698 | 1.88754290329093 | 0.00778570481085333 | 0.222988489458029 | 2.10870206625461 | 0.047244094488189 | FERMT2//ARFIP2//RHOF//RIC8B//BFSP1//NDFIP1//PARD6B//LRRK2//HIF1A//TMEM108//CCDC103//SEPT7//BSN//MAEA//ACTR2//MLPH//POLR2M//MAPRE1// |
| GO:0032279 | asymmetric synapse | Cellular component | 11 | 232 | 381 | 18698 | 2.32688478595348 | 0.00825094649687084 | 0.222988489458029 | 2.08349622905126 | 0.0288713910761155 | ACTR2//CHRM1//CHRNA3//FBXO45//CPEB3//GRIN2B//PALM//RTN4//TMEM108//ITGA8//BSN// |
| GO:0030055 | cell-substrate junction | Cellular component | 16 | 401 | 381 | 18698 | 1.95814924630681 | 0.00846110229006613 | 0.222988489458029 | 2.07257305453678 | 0.041994750656168 | ACTR3//ACTR2//FERMT2//FZD2//PDIA3//ITGA5//CD46//PARVA//RRAS//TLN1//YWHAB//YWHAZ//NUP214//ITGA8//DIXDC1//CD81// |
| GO:0098796 | membrane protein complex | Cellular component | 36 | 1166 | 381 | 18698 | 1.51521454329358 | 0.00858143582537265 | 0.222988489458029 | 2.06644004095477 | 0.094488188976378 | PYURF//SPCS2//TOMM40L//CPLX1//VAMP3//GOSR2//INSIG2//EMC8//EVC//NRBF2//GNAO1//GNAZ//SCN3B//SCN1A//SCN7A//ATP1B4//TRDN//LRRC38//KCNIP3//KCNIP2//ABCC8//CNTN2//KCNQ4//SSPN//ITGA5//TIMM23//ATP6V1G2//AP1S1//CHRNA3//GRIN2B//VWC2//SESTD1//BEST1//ITGA8//SKAP1//CD8A// |
| GO:0070161 | anchoring junction | Cellular component | 20 | 546 | 381 | 18698 | 1.79765990789613 | 0.00868000509071042 | 0.222988489458029 | 2.06148002011535 | 0.05249343832021 | CTNND2//JAM3//EIF4G2//CADM2//ACTR3//ACTR2//FERMT2//FZD2//PDIA3//ITGA5//CD46//PARVA//RRAS//TLN1//YWHAB//YWHAZ//NUP214//ITGA8//DIXDC1//CD81// |
| GO:0098984 | neuron to neuron synapse | Cellular component | 11 | 235 | 381 | 18698 | 2.29717987379237 | 0.00903769282545356 | 0.222988489458029 | 2.04394242362415 | 0.0288713910761155 | ACTR2//CHRM1//CHRNA3//FBXO45//CPEB3//GRIN2B//PALM//RTN4//TMEM108//ITGA8//BSN// |
| GO:0044459 | plasma membrane part | Cellular component | 75 | 2830 | 381 | 18698 | 1.30060376728527 | 0.009085677684553 | 0.222988489458029 | 2.04164267389416 | 0.196850393700787 | RHOH//CD81//ITGA5//NCAM1//CD8A//PPP3CA//FZD3//PPP3CB//SCN1A//UNC5A//EVC//FERMT2//MAEA//SLC26A1//CHRM1//CHRNA3//LRRC38//AGTR1//EFNA3//P2RX2//ATP1B4//CADM2//GPR3//GPR34//GRIN2B//SLC25A5//SLC39A2//IL4R//TRABD2B//CD46//MRC1//PALM//CLEC1A//PCDHB3//SLC5A3//ABCC8//CNTN2//TMPRSS2//TNFSF4//SSPN//TSPAN14//TSPAN18//TNFRSF8//GAL3ST1//P2RY14//SLC23A2//SH3YL1//EPS8L2//TLN1//KCNQ4//BEST1//EMP2//PRKAA1//PARD6B//CYP4F2//VAMP3//SEPT7//MAPRE1//LRRK2//GNAO1//GNAZ//SCN3B//SCN7A//TRDN//KCNIP3//KCNIP2//SLC27A4//ITGA8//NOS3//FBXO45//CPEB3//LRRTM1//TMEM108//SKAP1//VWC2// |
| GO:0034706 | sodium channel complex | Cellular component | 3 | 23 | 381 | 18698 | 6.40123245463882 | 0.010985072456318 | 0.256545574243577 | 1.95919707417585 | 0.0078740157480315 | SCN3B//SCN1A//SCN7A// |
| GO:0008076 | voltage-gated potassium channel complex | Cellular component | 6 | 92 | 381 | 18698 | 3.20061622731941 | 0.0113007794703856 | 0.256545574243577 | 1.94689160005531 | 0.015748031496063 | LRRC38//KCNIP3//KCNIP2//ABCC8//CNTN2//KCNQ4// |
| GO:0098793 | presynapse | Cellular component | 15 | 379 | 381 | 18698 | 1.94232647040492 | 0.0113489311073992 | 0.256545574243577 | 1.94504504032271 | 0.0393700787401575 | COPS5//STON2//FBXO45//CPLX1//AP1S1//LRRK2//CHRM1//ELK1//KCNIP3//FZD3//BSN//ABCC8//SCAMP1//ATP6V1G2//ZNF804A// |
| GO:0005912 | adherens junction | Cellular component | 19 | 528 | 381 | 18698 | 1.76599657997296 | 0.0123452194714661 | 0.271911372461266 | 1.90850118484105 | 0.0498687664041995 | EIF4G2//CADM2//ACTR3//ACTR2//FERMT2//FZD2//PDIA3//ITGA5//CD46//PARVA//RRAS//TLN1//YWHAB//YWHAZ//NUP214//ITGA8//DIXDC1//CD81//CTNND2// |
| GO:0044444 | cytoplasmic part | Cellular component | 218 | 9626 | 381 | 18698 | 1.11142667523925 | 0.0133619788426028 | 0.286948495644895 | 1.87412922024495 | 0.572178477690289 | PPP1R2//CARHSP1//CCDC103//SEPT7//TIMM23//PYURF//EMC8//ZBTB6//TOMM34//AKAP10//LRRK2//STARD4//PPTC7//DLAT//ELK1//DDAH1//MYCBP//SLC25A5//ACADL//ME2//UCHL5//MRPS33//PMAIP1//PPP3CA//RFK//CREBZF//CEP85//ABCC8//YWHAB//YWHAZ//OGT//NFS1//SH3BP5//BAG5//ACTR3//ACTR2//FAM13A//CALCOCO2//CDKN1B//TRDN//CPLX1//RALBP1//FERMT2//COPS5//NISCH//AP1S1//UBXN2B//CTH//PNPLA5//RNF217//ARHGAP36//UBXN2A//DPYS//EIF4A2//EIF4G2//EMP2//EPAS1//HIPK1//ALAD//FABP2//MAPRE1//RRP1B//MGRN1//ARFIP2//FTSJ1//SULT4A1//FBXL3//PTPN18//GNAZ//WDR91//KCNIP3//HIF1A//IDI1//KIF2A//KRT2//TNFAIP8L3//RHOH//SMAD1//NCAM1//NOS3//SPRN//PABPC3//PAX5//ABHD5//RLIM//UPB1//ATP6V1G2//POLR2E//RHOF//SHQ1//RIC8B//NUDT15//VPS53//PPP3CB//PRKAA1//MAP7D1//PARVA//PRKAR2B//BRK1//MAPK4//KLHL7//GRIPAP1//TULP4//PSMD7//KLHL14//WDFY1//PEX5//TP53INP2//PCTP//RPE//SFRP1//EPS8L2//SKP1//HSPA13//TLN1//UBE2D3//UBE2E1//UCK2//BEST1//RPAP3//ZNF322//PIP4K2C//AGBL2//NUP214//TNKS2//KRTAP1-1//CASP7//MEX3B//RNF135//PARD6B//CREB3L3//PARG//STON2//DIXDC1//SKAP1//ALDH1A2//KBTBD6//SYS1//CH25H//BUB3//COPS2//VAMP3//PAXBP1//ARHGEF6//BAG2//GOSR2//OXSR1//KDELR3//PDIA3//LRRTM1//ITGA5//MEST//DNAJB12//JPH1//RTN4//SLC35A2//MOGAT2//TXNDC5//CAMLG//ITGA8//AOC3//NUCB1//DBI//NRAS//NDFIP1//JAM3//GPD2//NRBF2//SLC39A2//PALM//CXXC4//FAM168B//MLPH//SMPD1//NPTX1//SSPN//PNRC1//LARP1//RBM3//AADAT//GOLGA7B//ST3GAL3//GAL3ST1//SPCS2//EBI3//GPX8//SLC27A4//SLC35G1//CYP4F3//INSIG2//OTOR//CYP4F2//CD46//CTNS//MAEA//BMP10//SCN3B//SCN1A//POLR2M//BFSP1//DRAM1//SNX31//TMEM108//AVL9//VLDLR//MRPL50//SCAMP1//ABCA13//FZD2//TSPAN14//BSN//SNN//LETM2//RBFOX1//MRC1//TOMM40L//FERMT3//SKIL// |
| GO:0030018 | Z disc | Cellular component | 7 | 125 | 381 | 18698 | 2.7482624671916 | 0.0141741895341464 | 0.29696655633736 | 1.84850176424534 | 0.0183727034120735 | BMP10//PPP3CA//PPP3CB//PARVA//SCN3B//JPH1//SCN1A// |
| GO:0030424 | axon | Cellular component | 19 | 538 | 381 | 18698 | 1.73317136473182 | 0.0147776644175062 | 0.302238422253282 | 1.83039419995617 | 0.0498687664041995 | LRRK2//LRRTM1//ZNF804A//SCN1A//CNTN2//CPLX1//AP1S1//CHRM1//ELK1//KCNIP3//RTN4//HIF1A//TMEM108//FAM168B//CADM2//PALM//GRIPAP1//FZD3//BSN// |
| GO:0034705 | potassium channel complex | Cellular component | 6 | 99 | 381 | 18698 | 2.97431002942814 | 0.0157929144620431 | 0.315491012160349 | 1.80153771685776 | 0.015748031496063 | LRRC38//KCNIP3//KCNIP2//ABCC8//CNTN2//KCNQ4// |
| GO:0044297 | cell body | Cellular component | 18 | 513 | 381 | 18698 | 1.72196896440577 | 0.0184137938594131 | 0.359487475573542 | 1.73485672304076 | 0.047244094488189 | CPLX1//CHRNA3//LRRK2//ELK1//PRKAR2B//RTN4//KLHL14//PURA//SCN1A//CNTN2//FZD3//POLR2M//ZNF804A//UNC5A//CTNND2//ITGA8//GNAO1//GNAZ// |
| GO:0030017 | sarcomere | Cellular component | 9 | 197 | 381 | 18698 | 2.24205603741157 | 0.019953847770063 | 0.36720821427136 | 1.6999733453819 | 0.0236220472440945 | FERMT2//POLR2M//BMP10//PPP3CA//PPP3CB//PARVA//SCN3B//JPH1//SCN1A// |
| GO:0031256 | leading edge membrane | Cellular component | 8 | 165 | 381 | 18698 | 2.37944802354251 | 0.020030283945663 | 0.36720821427136 | 1.69831289418318 | 0.020997375328084 | FERMT2//SH3YL1//ITGA5//EPS8L2//TLN1//UNC5A//PALM//ITGA8// |
| GO:0005885 | Arp2/3 protein complex | Cellular component | 2 | 11 | 381 | 18698 | 8.92293008828442 | 0.0201688577551715 | 0.36720821427136 | 1.6953186969619 | 0.005249343832021 | ACTR3//ACTR2// |
| GO:0030863 | cortical cytoskeleton | Cellular component | 6 | 105 | 381 | 18698 | 2.80434945631796 | 0.0205192017287838 | 0.36720821427136 | 1.68783953885885 | 0.015748031496063 | MLPH//POLR2M//MAPRE1//BSN//MAEA//ACTR2// |
| GO:0043025 | neuronal cell body | Cellular component | 16 | 449 | 381 | 18698 | 1.74881480572167 | 0.0223516113495048 | 0.391837431616829 | 1.65069116268985 | 0.041994750656168 | UNC5A//LRRK2//CTNND2//ITGA8//CPLX1//CHRNA3//ELK1//PRKAR2B//RTN4//KLHL14//PURA//SCN1A//CNTN2//FZD3//POLR2M//ZNF804A// |
| GO:0005802 | trans-Golgi network | Cellular component | 9 | 202 | 381 | 18698 | 2.18655960084197 | 0.0230282275192411 | 0.395624948780562 | 1.63773948835956 | 0.0236220472440945 | AP1S1//ARFIP2//VPS53//SYS1//VAMP3//LRRK2//NUCB1//RBFOX1//SCAMP1// |
| GO:0044306 | neuron projection terminus | Cellular component | 7 | 139 | 381 | 18698 | 2.47145905322986 | 0.0239056435548102 | 0.39773133277839 | 1.62149956033294 | 0.0183727034120735 | CHRM1//ELK1//KCNIP3//CPLX1//AP1S1//LRRK2//BSN// |
| GO:0032991 | protein-containing complex | Cellular component | 121 | 5071 | 381 | 18698 | 1.17101360160782 | 0.024076867641998 | 0.39773133277839 | 1.61840001468508 | 0.31758530183727 | TTK//BUB3//FERMT3//PURA//EPAS1//HIF1A//SMAD1//PSMD7//ALAD//COPS5//ACTR3//ACTR2//PRKAR2B//COPS2//EIF4A2//EIF4G2//PFDN1//CGNL1//RLIM//YWHAB//CPEB3//TRDN//JPH1//UBXN10//NUP214//SEPT7//BRK1//SKP1//LARP1//FERMT2//KCNIP3//MAEA//P2RX2//GPR160//IL4R//VLDLR//SLC25A5//C2ORF49//U2AF1//RPAP3//KIF2A//VPS53//LRRK2//SSPN//ITGA5//ITGA8//PYURF//SPCS2//TOMM40L//CPLX1//VAMP3//GOSR2//INSIG2//EMC8//EVC//MRPL50//MRPS33//DLAT//PPIH//SNRPA//LSM11//ATP6V1G2//AP1S1//NRBF2//RPP14//RRP1B//UCHL5//ACTL6A//PNRC1//CARHSP1//MEX3B//CHRNA3//SKAP1//CD8A//RBM3//GNAO1//GNAZ//SCN3B//SCN1A//SCN7A//TIMM23//ATP1B4//LRRC38//KCNIP2//ABCC8//CNTN2//KCNQ4//UBE4B//RNF217//FBXL3//UBE2E1//GOLGA7B//PGGT1B//GPD2//BEST1//PPP1R2//PPP3CA//PPP3CB//PRDM4//PAXIP1//ZFP64//FBXO45//ZSWIM6//KLHL7//KLHL14//KBTBD6//CDKN1B//GRIN2B//VWC2//POLR2E//POLR2M//OGT//SESTD1//PRKAA1//AKAP10//NAT9//FOXP3//ASF1B//KMT2E//PEX5//EPS8L2// |
| GO:0032591 | dendritic spine membrane | Cellular component | 2 | 13 | 381 | 18698 | 7.55017161316374 | 0.0278474899387409 | 0.438827091795615 | 1.5552139442921 | 0.005249343832021 | PALM//ITGA8// |
| GO:0043218 | compact myelin | Cellular component | 2 | 13 | 381 | 18698 | 7.55017161316374 | 0.0278474899387409 | 0.438827091795615 | 1.5552139442921 | 0.005249343832021 | JAM3//PLLP// |
| GO:0016020 | membrane | Cellular component | 211 | 9426 | 381 | 18698 | 1.09856358661724 | 0.0280971944688694 | 0.438827091795615 | 1.55133704264002 | 0.553805774278215 | EBI3//TRDN//SLC26A1//SLC27A4//PTPRT//NISCH//AKAP10//CHRM1//CHRNA3//LRRK2//FAM168B//LYPD6//CTNS//ABCA13//SLC35G1//AGTR1//EFNA3//EMP2//EWSR1//P2RX2//MGRN1//ARFIP2//FZD2//CADM2//SYT14//GPR160//GNAO1//GNAZ//GRIN2B//SLC39A2//KCNIP3//KCNIP2//IL4R//ITGA5//TNFAIP8L3//RHOH//ERVFRD-1//MARK1//CD46//MRC1//NCAM1//NOS3//NPTX1//NRAS//SPRN//PALM//CLDN18//RHOF//ENOX1//RALGPS2//RIC8B//PPP3CB//PARVA//PRKAR2B//SCN3B//KMT2E//KLHL7//JPH1//RTN4//SEMA4G//RRAS//BFSP1//SCN1A//SCN7A//ANO3//SFRP1//EPS8L2//SLC5A3//SMPD1//HSPA13//ABCC8//CNTN2//TMPRSS2//TNFSF4//UBE2D3//VLDLR//BEST1//FZD3//TSPAN14//JAM3//PARD6B//OGT//ITGA8//TMEFF1//ACTL6A//SKAP1//AOC3//TNFSF14//UNC5A//KCNQ4//ZNF804A//CD8A//VAMP3//TNFRSF8//SCAMP1//CD81//P2RY14//SLC23A2//PYURF//KDELR3//CYP4F3//MEST//INSIG2//OTOR//KLHL14//MOGAT2//CREB3L3//CYP4F2//CH25H//GOSR2//SPCS2//KRT2//EVPLL//PPP3CA//SSPN//DNAJB12//ACADL//PRKAA1//SEPT7//MAPRE1//VPS53//TMEM108//NDFIP1//CALCOCO2//AP1S1//DRAM1//WDR91//GRIPAP1//PDIA3//YWHAB//YWHAZ//ATP1B4//SMAD1//TIMM23//LETM2//GPD2//SLC25A5//MRPS33//MRPL50//TOMM34//PMAIP1//SNN//GOLGA7B//ST3GAL3//SLC35A2//TNKS2//GAL3ST1//EVC//FERMT2//SH3YL1//TLN1//PEX5//FBXO45//CPEB3//LRRTM1//NUCB1//SYS1//PLLP//ATP6V1G2//SMIM12//TM4SF18//TMEM132E//TEDDM1//RNF217//KIAA1324L//AVL9//EPHX4//FAM19A5//SLC46A3//C16ORF54//RNF149//IGSF1//TMEM151B//GPX8//TMEM248//SLC35E3//PCDHB3//SLC35E2B//ADAM33//CAMLG//TMEM246//PPP1R3F//MAEA//LRRC38//GPR3//GPR34//TRABD2B//CLEC1A//TSPAN18//TOMM40L//CPLX1//VWC2//SESTD1//NRBF2//EMC8//ACTR3//ACTR2//RALBP1//PNPLA5//EIF4G2//FAM83B//LARP1//KIF2A//PSMD7//PTBP1//RASL11B//TTK//PIP4K2C//FERMT3//STON2//BAG5// |
| GO:0033267 | axon part | Cellular component | 13 | 353 | 381 | 18698 | 1.80733569776866 | 0.0296334816044013 | 0.449366026366482 | 1.52821732075241 | 0.0341207349081365 | LRRK2//LRRTM1//ZNF804A//SCN1A//CNTN2//CPLX1//AP1S1//CHRM1//ELK1//KCNIP3//HIF1A//TMEM108//RTN4// |
| GO:0032588 | trans-Golgi network membrane | Cellular component | 5 | 85 | 381 | 18698 | 2.88683032268025 | 0.0298182345784511 | 0.449366026366482 | 1.52551807300688 | 0.0131233595800525 | AP1S1//ARFIP2//VPS53//SYS1//VAMP3// |
| GO:0044449 | contractile fiber part | Cellular component | 9 | 214 | 381 | 18698 | 2.06394878210317 | 0.0317614851637364 | 0.470398547511199 | 1.49809919820542 | 0.0236220472440945 | BMP10//PPP3CA//PPP3CB//PARVA//SCN3B//JPH1//SCN1A//FERMT2//POLR2M// |
| GO:0005887 | integral component of plasma membrane | Cellular component | 45 | 1678 | 381 | 18698 | 1.31610560003003 | 0.0347006696261836 | 0.489900322812675 | 1.45966214445551 | 0.118110236220472 | SCN3B//SCN1A//SCN7A//ITGA5//ITGA8//ATP1B4//CHRNA3//LRRC38//KCNIP3//KCNIP2//ABCC8//CNTN2//KCNQ4//GRIN2B//VWC2//MAEA//SLC26A1//CHRM1//AGTR1//EFNA3//P2RX2//CADM2//GPR3//GPR34//SLC25A5//SLC39A2//IL4R//TRABD2B//CD46//MRC1//PALM//CLEC1A//PCDHB3//SLC5A3//TMPRSS2//TNFSF4//SSPN//TSPAN14//TSPAN18//CD8A//TNFRSF8//GAL3ST1//CD81//P2RY14//SLC23A2// |
| GO:0071944 | cell periphery | Cellular component | 131 | 5614 | 381 | 18698 | 1.14516763958121 | 0.0356422617384196 | 0.489900322812675 | 1.44803474488912 | 0.343832020997375 | EBI3//TRDN//SLC26A1//SLC27A4//PTPRT//NISCH//AKAP10//CHRM1//CHRNA3//LRRK2//FAM168B//LYPD6//CTNS//ABCA13//SLC35G1//AGTR1//EFNA3//EMP2//EWSR1//P2RX2//MGRN1//ARFIP2//FZD2//CADM2//SYT14//GPR160//GNAO1//GNAZ//GRIN2B//SLC39A2//KCNIP3//KCNIP2//IL4R//ITGA5//TNFAIP8L3//RHOH//ERVFRD-1//MARK1//CD46//MRC1//NCAM1//NOS3//NPTX1//NRAS//SPRN//PALM//CLDN18//RHOF//ENOX1//RALGPS2//RIC8B//PPP3CB//PARVA//PRKAR2B//SCN3B//KMT2E//KLHL7//JPH1//RTN4//SEMA4G//RRAS//BFSP1//SCN1A//SCN7A//ANO3//SFRP1//EPS8L2//SLC5A3//SMPD1//HSPA13//ABCC8//CNTN2//TMPRSS2//TNFSF4//UBE2D3//VLDLR//BEST1//FZD3//TSPAN14//JAM3//PARD6B//OGT//ITGA8//TMEFF1//ACTL6A//SKAP1//AOC3//TNFSF14//UNC5A//KCNQ4//ZNF804A//CD8A//VAMP3//TNFRSF8//SCAMP1//CD81//P2RY14//SLC23A2//FERMT2//NDFIP1//KRT2//EVPLL//PPP3CA//SSPN//BSN//MAEA//LRRC38//ATP1B4//GPR3//GPR34//SLC25A5//TRABD2B//CLEC1A//PCDHB3//TSPAN18//GAL3ST1//PRKAA1//CYP4F2//SEPT7//MAPRE1//VWC2//SH3YL1//TLN1//FBXO45//CPEB3//LRRTM1//TMEM108//EVC//ACTR2//MLPH//POLR2M// |
| GO:0030016 | myofibril | Cellular component | 9 | 219 | 381 | 18698 | 2.01682666379031 | 0.036001800153866 | 0.489900322812675 | 1.44367578319536 | 0.0236220472440945 | FERMT2//POLR2M//BMP10//PPP3CA//PPP3CB//PARVA//SCN3B//JPH1//SCN1A// |
| GO:0031011 | Ino80 complex | Cellular component | 2 | 15 | 381 | 18698 | 6.54348206474191 | 0.0365001404656708 | 0.489900322812675 | 1.43770546421891 | 0.005249343832021 | UCHL5//ACTL6A// |
| GO:0033202 | DNA helicase complex | Cellular component | 2 | 15 | 381 | 18698 | 6.54348206474191 | 0.0365001404656708 | 0.489900322812675 | 1.43770546421891 | 0.005249343832021 | UCHL5//ACTL6A// |
| GO:0033268 | node of Ranvier | Cellular component | 2 | 15 | 381 | 18698 | 6.54348206474191 | 0.0365001404656708 | 0.489900322812675 | 1.43770546421891 | 0.005249343832021 | SCN1A//CNTN2// |
| GO:0031461 | cullin-RING ubiquitin ligase complex | Cellular component | 8 | 188 | 381 | 18698 | 2.08834533981125 | 0.0391837463479184 | 0.512451620280769 | 1.40689404357236 | 0.020997375328084 | FBXO45//FBXL3//SKP1//ZSWIM6//KLHL7//KLHL14//KBTBD6//CDKN1B// |
| GO:0005776 | autophagosome | Cellular component | 5 | 92 | 381 | 18698 | 2.66718018943284 | 0.0399205023890203 | 0.512451620280769 | 1.39880400186847 | 0.0131233595800525 | CALCOCO2//LRRK2//NRBF2//TP53INP2//PIP4K2C// |
| GO:0043679 | axon terminus | Cellular component | 6 | 123 | 381 | 18698 | 2.39395685295436 | 0.04018148761276 | 0.512451620280769 | 1.39597398869147 | 0.015748031496063 | CPLX1//AP1S1//LRRK2//CHRM1//ELK1//KCNIP3// |
| GO:0031226 | intrinsic component of plasma membrane | Cellular component | 46 | 1741 | 381 | 18698 | 1.29666933505799 | 0.0407930173514531 | 0.512451620280769 | 1.3894141698822 | 0.120734908136483 | MAEA//SLC26A1//CHRM1//CHRNA3//LRRC38//AGTR1//EFNA3//P2RX2//ATP1B4//CADM2//GPR3//GPR34//GRIN2B//SLC25A5//SLC39A2//IL4R//TRABD2B//CD46//MRC1//PALM//CLEC1A//PCDHB3//SLC5A3//ABCC8//CNTN2//TMPRSS2//TNFSF4//SSPN//TSPAN14//TSPAN18//CD8A//TNFRSF8//GAL3ST1//CD81//P2RY14//SLC23A2//SCN3B//SCN1A//SCN7A//ITGA5//ITGA8//KCNIP3//KCNIP2//KCNQ4//VWC2//UNC5A// |
| GO:0000800 | lateral element | Cellular component | 2 | 16 | 381 | 18698 | 6.13451443569554 | 0.0411631685673726 | 0.512451620280769 | 1.38549120246254 | 0.005249343832021 | FAM9A//FAM9C// |
| GO:0043292 | contractile fiber | Cellular component | 9 | 230 | 381 | 18698 | 1.92036973639165 | 0.0466606445172318 | 0.57259276629003 | 1.33104926664934 | 0.0236220472440945 | BMP10//PPP3CA//PPP3CB//PARVA//SCN3B//JPH1//SCN1A//FERMT2//POLR2M// |
| GO:1902494 | catalytic complex | Cellular component | 38 | 1417 | 381 | 18698 | 1.31608496009276 | 0.0499663048329421 | 0.596339555200706 | 1.3013227668272 | 0.0997375328083989 | U2AF1//UCHL5//ACTL6A//GNAO1//GNAZ//SPCS2//GPD2//DLAT//UBE4B//RNF217//FBXL3//UBE2E1//PYURF//GOLGA7B//PGGT1B//FBXO45//SKP1//ZSWIM6//KLHL7//KLHL14//KBTBD6//CARHSP1//PPP1R2//PPP3CA//PPP3CB//PRDM4//PAXIP1//ZFP64//POLR2E//PRKAA1//NRBF2//ATP1B4//OGT//RPP14//PSMD7//CDKN1B//POLR2M//ALAD// |
| GO:0044425 | membrane part | Cellular component | 154 | 6780 | 381 | 18698 | 1.1147082278432 | 0.049984223485973 | 0.596339555200706 | 1.30116705034728 | 0.404199475065617 | PYURF//SLC27A4//KDELR3//SLC35G1//CYP4F3//MEST//INSIG2//JPH1//OTOR//RTN4//KLHL14//MOGAT2//CREB3L3//CYP4F2//CH25H//GOSR2//SPCS2//AP1S1//VLDLR//TRDN//SLC26A1//TOMM34//PTPRT//SMIM12//CHRNA3//TM4SF18//TMEM132E//TEDDM1//FAM168B//LETM2//CTNS//RNF217//ABCA13//AGTR1//EMP2//EVC//KIAA1324L//P2RX2//AVL9//EPHX4//FZD2//SYT14//FAM19A5//GPR160//SLC46A3//C16ORF54//RNF149//SLC25A5//LRRTM1//IGSF1//ERVFRD-1//SMAD1//TMEM151B//NCAM1//GPX8//PLLP//CLDN18//TMEM248//DRAM1//SLC35E3//SCN3B//PCDHB3//SEMA4G//ANO3//SFRP1//ST3GAL3//SLC5A3//TMEM108//SLC35E2B//SLC35A2//BEST1//FZD3//ADAM33//NDFIP1//CAMLG//SNN//JAM3//TMEM246//TMEFF1//AOC3//TNFSF14//PPP1R3F//UNC5A//KCNQ4//VAMP3//SCAMP1//P2RY14//SLC23A2//LYPD6//EFNA3//SPRN//CNTN2//LRRK2//RHOH//CD81//ITGA5//CD8A//PPP3CA//PPP3CB//SCN1A//CD46//TOMM40L//CPLX1//EMC8//ATP6V1G2//MAEA//CHRM1//LRRC38//ATP1B4//CADM2//GPR3//GPR34//GRIN2B//SLC39A2//IL4R//TRABD2B//MRC1//PALM//CLEC1A//ABCC8//TMPRSS2//TNFSF4//SSPN//TSPAN14//TSPAN18//TNFRSF8//GAL3ST1//SYS1//DNAJB12//FERMT2//WDR91//NUCB1//NRBF2//SH3YL1//EPS8L2//TLN1//ITGA8//TIMM23//GNAO1//GNAZ//SCN7A//KCNIP3//KCNIP2//PRKAA1//PARD6B//SEPT7//MAPRE1//PRKAR2B//NOS3//FBXO45//CPEB3//SKAP1//VWC2//SESTD1// |
